# Bronchogenic cyst of the floor of mouth combined with ankyloglossia: an extremely rare presentation of rare anomaly

**DOI:** 10.1093/jscr/rjab211

**Published:** 2021-05-27

**Authors:** Nuttadon Wongprakob, Suthida Sae-Guay, Chairat Burusapat, Kittisak Wongchansom, Nutthapong Wanichjaroen

**Affiliations:** Division of Plastic and Reconstructive Surgery, Department of Surgery, Phramongkutklao Hospital, Bangkok, Thailand; Division of Pediatric Surgery, Department of Surgery, Phramongkutklao Hospital and Phramongkutklao College of Medicine, Bangkok, Thailand; Division of Plastic and Reconstructive Surgery, Department of Surgery, Phramongkutklao Hospital and Phramongkutklao College of Medicine, Bangkok, Thailand; Department of Pathology and Laboratory Medicine, Phramongkutklao Hospital, Bangkok, Thailand; Division of Plastic and Reconstructive Surgery, Department of Surgery, Phramongkutklao Hospital and Phramongkutklao College of Medicine, Bangkok, Thailand

## Abstract

Bronchogenic cyst (BC), cyst lined by respiratory epithelium, is uncommon congenital anomaly of bronchial tree. Intraoral BC is extremely rare lesions. Here, we report the unusual presentation of 2-year-old boy with symptomatic cystic lesion at floor of month combined with ankyloglossia. The operation was performed under general anesthesia. Frenotomy was performed. Complete cystic removal was successful with minimal leakage of cyst wall. Sclerosing agent was injected at surgical site to prevent the residual undetected cystic malformation. Pathological examination was demonstrated a unicystic lesion lined by ciliated pseudostratified columnar and cuboidal cells. The final diagnosis was bronchogenic cyst. No postoperative complication was found. The long-term course was uneventful with no signs of recurrence at 1 year. To our best knowledge, a rare example of BC at the floor of mouth combined with ankyloglossia has never been reported.

## INTRODUCTION

Bronchogenic cyst (BC), cyst lined by respiratory epithelium, is uncommon congenital anomaly of bronchial tree. They are usually located in the mediastinum near the tracheobronchial tree (paratracheal, carinal or hilar) or adjacent to the esophageal wall [1]. Intrapericardial, transdiaphragmatic, intra-abdominal and subcutaneous sites have been reported [1–5]. Intraoral BC is extremely rare lesions [6]. Here, we report the unusual presentation of 2-year-old boy with symptomatic cystic lesion at floor of month combined with ankyloglossia, which has not previously been reported in English literature as the combined anomalies.

**Figure 1:**
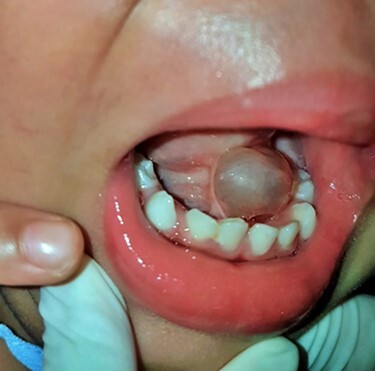
Two-year-old boy with cystic lesion at floor of month combined with ankyloglossia.

**Figure 2:**
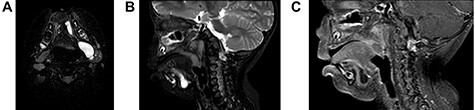
(**A**) On axial view, MRI demonstrated multi-lobulated cystic mass, which showed hypersignal intensity on T2 weighted image involving left sublingual, submandibular, pharyngeal and parapharyngeal spaces; (**B**) On sagittal view, MRI demonstrated multi-lobulated cystic mass, which showed hypersignal intensity on T2 weighted image involving left sublingual and submandibular spaces; (**C**) On sagittal view, MRI demonstrated multi-lobulated cystic mass, which showed hyposignal intensity on T1 fat suppression with gadolinium involving left sublingual and submandibular spaces.

## CASE REPORT

A 2-year-old boy presented with a slow growing mass under the tongue for 6 months. He was born without any anomaly. The development status was within average except delay speech due to the mass at floor of mouth. The physical examination revealed left sublingual cystic lesion measuring 1.0 × 1.0 cm. Ankyloglossia was found (Fig. 1). Cervical lymph nodes could not be palpated. Magnetic resonance imaging (MRI) demonstrated multi-lobulated cystic mass, size about 3.15 × 2.1 × 2.9 cm, which showed hypersignal intensity on T2 weighted image, hyposignal intensity on T1 weighted image, T1 fat suppression, T1 fat suppression with gadolinium involving left sublingual, submandibular, pharyngeal and parapharyngeal spaces (Fig. 2A–C). The provisional diagnosis was lymphatic or venous malformation.

**Figure 3:**
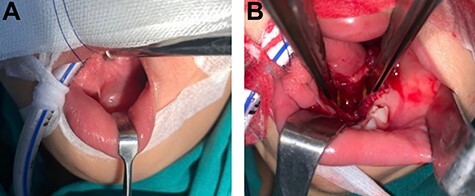
(**A**) The operation was performed under general anesthesia; (**B**) Frenotomy was performed then cystic mass was removed and it showed leave cavity on sublingual and submandibular spaces.

**Figure 4:**
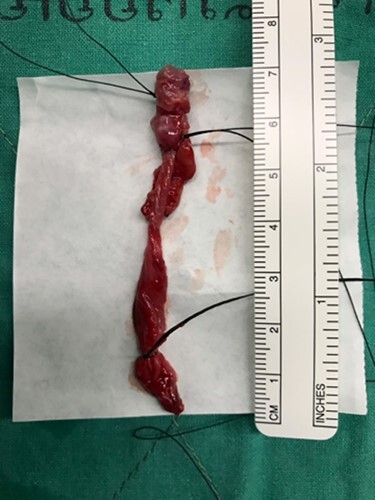
The specimen showed a multi-lobulated cystic cavity with leakage of fluid content.

**Figure 5:**
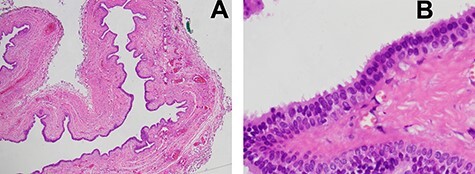
(**A**) Microscopic examination was demonstrated a unicystic lesion lined by ciliated pseudostratified columnar and cuboidal cells (low-power field); (**B**) Microscopic examination was demonstrated ciliated pseudostratified columnar and cuboidal cells lining. Goblet cells were not discerned. Subepithelial stroma revealed only fibrovascular tissue (high-power field).

The operation was performed under general anesthesia. Frenotomy was performed. Complete cystic removal was successful with minimal leakage of cyst wall (Fig. 3A and B). However, sclerosing agent was injected at surgical site to prevent the residual undetected cystic malformation. Macroscopic examination of the specimen showed a multi-lobulated cystic cavity with leakage of fluid content (Fig. 4). Pathological examination was demonstrated a unicystic lesion lined by ciliated pseudostratified columnar and cuboidal cells. No goblet cells were discerned. Subepithelial stroma revealed fibrovascular tissue. There was no other mesenchymal component such as smooth muscle bundles and cartilages (Fig. 5A–C). The final diagnosis was bronchogenic cyst. No postoperative complication was found. The long-term course was uneventful with no signs of recurrence at 1 year.

## DISCUSSION

BC, rare developmental anomalies, is lined by respiratory epithelium, i.e. ciliated, pseudostratified squamous epithelium, comprising mucus-secreting cells, smooth muscle cells and cartilaginous tissue. They are usually located in the mediastinum adjacent to the tracheobronchial tree or esophageal wall [1]. Combined other anomaly with BC is extremely presented. To our best knowledge, a rare example of BC at the floor of mouth combined with ankyloglossia has never been reported.

The pathogenesis of BC has not yet been demonstrated, however, several hypotheses have been proposed in the literature. BC may result from an early aberrant nodule that becomes detached from the primitive tracheobronchial tree or may be derived from cells of the esophagotracheal ridge, forming an isolated bronchial structure [7].

Mediastinal BC is often discovered at birth, usually asymptomatic and accidental discovered later in adult. When BC increases in size, it can be complicated by upper airway obstruction, causing feeding and even respiratory difficulties, infection and possibly abscess formation [8]. However, intraoral BC was reported in neonate with a congenital sublingual cystic lesion and impeding breast-feeding [9].

Our patient presented a cystic lesion at floor of mount that interfered speech and breast-feeding. Moreover, it also associated tongue tie. The differential diagnosis of cystic lesion at the tongue include lymphoepithelial cyst, lingual thyroid, thyroglossal duct cyst, epidermoid cyst, dermoid cyst, mucocele, mucous retention cyst, cystic hygroma and hemangioma [10]. The distinction between lymphatic malformation and BC is important, as the first-line treatments are very different: injection of sclerosing agents into the lesion in the case of lymphatic malformation and primary surgical enucleation for lingual choristomas (BC and gastrointestinal duplications), even hemangioma and sialocele [11]. Furthermore, no patient was diagnosed correctly prior to surgical excision [12].

Investigation for accurate diagnosis is important. Computed tomography (CT) and/or MRI are performed. CT of BC typically shows sharply marginated mediastinal masses of soft-tissue or water attenuation. Sometime, CT can be confused with other lesions; MRI can be useful for elucidating the cystic nature of these lesions [13].

MRI demonstrated multi-lobulated cystic mass, which showed hypersignal intensity on T2 weighted image, hyposignal intensity on T1 weighted image. Contrast to some literature that MRI revealed a homogeneous, unilocular cystic lesion with hypersignal intensity on T2 and hyposignal intensity on T1 with contrast enhancement after gadolinium infusion [7]. According to the imaging feature demonstrate likely lymphatic malformation, although final diagnosis was a cyst lined by respiratory and squamous epithelium or BC.

Malignant transformation of BC to adenocarcinoma has been reported in adults with untreated chronic lingual cysts [14]. However, incidence and prognosis are still inconclusive.

Surgically enucleated is recommended for diagnosis, treatment and prevention of possible complications. Surgical enucleation should be performed at the time of the first complications or at the age of 1 year, constituting a balance between the risks related to general anesthesia [11, 15].

## CONCLUSION

Bronchogenic cyst at floor of mouth with ankyloglossia is an extremely rare anomaly. MRI should be the investigation in the presence of a cystic lesion of floor of the mouth in children in order to evaluate the precise anatomical relations, which helps to guide the diagnosis. Surgical excision is recommended. Bronchogenic cyst should be kept in mind in cystic lesion of floor of month.

## CONFLICT OF INTEREST STATEMENT

None declared.

## FUNDING

None.
